# A Case Study of Waste Scrap Tyre-Derived Carbon Black Tested for Nitrogen, Carbon Dioxide, and Cyclohexane Adsorption

**DOI:** 10.3390/molecules25194445

**Published:** 2020-09-27

**Authors:** Zuzana Jankovská, Marek Večeř, Ivan Koutník, Lenka Matějová

**Affiliations:** 1 Faculty of Materials Science and Technology, Department of Chemistry, VŠB—Technical University of Ostrava, 17. listopadu 15/2172, 70800 Ostrava, Czech Republic; marek.vecer@vsb.cz (M.V.); ivan.koutnik@vsb.cz (I.K.); 2 Institute of Environmental Technology, VŠB—Technical University of Ostrava, 17. listopadu 15/2172, 70800 Ostrava, Czech Republic; lenka.matejova@vsb.cz

**Keywords:** sorption, CO_2_, cyclohexane, N_2_, waste scrap tyres, carbon black

## Abstract

Waste scrap tyres were thermally decomposed at the temperature of 600 °C and heating rate of 10 °C·min^−1^. Decomposition was followed by the TG analysis. The resulting pyrolytic carbon black was chemically activated by a KOH solution at 800 °C. Activated and non-activated carbon black were investigated using high pressure thermogravimetry, where adsorption isotherms of N_2_, CO_2_, and cyclohexane were determined. Isotherms were determined over a wide range of pressure, 0.03–4.5 MPa for N_2_ and 0.03–2 MPa for CO_2_. In non-activated carbon black, for the same pressure and temperature, a five times greater gas uptake of CO_2_ than N_2_ was determined. Contrary to non-activated carbon black, activated carbon black showed improved textural properties with a well-developed irregular mesoporous-macroporous structure with a significant amount of micropores. The sorption capacity of pyrolytic carbon black was also increased by activation. The uptake of CO_2_ was three times and for cyclohexane ten times higher in activated carbon black than in the non-activated one. Specific surface areas evaluated from linearized forms of Langmuir isotherm and the BET isotherm revealed that for both methods, the values are comparable for non-activated carbon black measured by CO_2_ and for activated carbon black measured by cyclohexane. It was found out that the N_2_ sorption capacity of carbon black depends only on its specific surface area size, contrary to CO_2_ sorption capacity, which is affected by both the size of specific surface area and the nature of carbon black.

## 1. Introduction

Due to their excellent properties, polymers have become irreplaceable in our lives, and their production and consumption is growing year by year. As a consequence, the amount of waste polymers produced is growing as well. When it comes to environmental protection and sustainable development, upgrading polymer waste to usable material is a key issue. Unfortunately, waste disposal and polymer-based waste incineration still remain the most common ways of dealing with problems regarding potentially hazardous material disposal today [1] of this waste.

In our industrial world, approximately one scrap tyre (ST) is produced per person every year [2]. It means that in the European Union 15 million tons of ST are disposed of every year. This rapid trend, along with the fact that the majority of these tyres are stockpiled in landfills, presents one of the greatest. For this reason, great attention is currently being paid to ST pyrolysis.

Pyrolysis may be a prospective way of reducing the amount of ST, or even of converting them into valuable material. Generally speaking, pyrolysis is thermal degradation (without oxygen), which yields solid, liquid, and gas products that may be further used. Typical yields of ST pyrolysis depend on process conditions (temperature, heating rate, and inert gas flow rate) and used facilities, and are as follows: 33–38 wt% of solid product, 38–55 wt% of liquid product, and 10–30 wt% of gas product [3]. The pyrolysis process is widely used, producing a solid residue with enhanced properties, the so-called pyrolytic carbon black (CBp). As a raw material, ST consists of fewer chemical components, has greater gross calorific value than coal [4], and is widely available, cheap, and suitable for recycling as a material rich in carbon.

The CBp produced by ST pyrolysis could become a marketable and commercially usable product, as long as its properties are similar to the manufactured carbon blacks (CBs). CBp produced by pyrolysis of waste ST can be used in many different ways, e.g., as sorbents, pigments, in rubber or battery production, etc. [5,6,7,8,9,10].

Adsorption is coming to be regarded as a practicable separation method for purification or bulk separation in newly developed material production processes, e.g., high-tech materials and biochemical and biomedical products. Surface characteristics and pore structures of adsorbents are the main properties in determining adsorption equilibrium and rate properties, which are needed for plant design. New adsorbents are being developed all the time, introducing new applications for adsorption technology [6,11].

One of the methods suitable for adsorption measurement and characterization of specific surface area is the gravimetric sorption method of gas sorption. The principle of this method [12] consists of exact measurements of mass difference at constant pressure steps and constant temperature. Gravimetric methods are great at high precision and sensitivity, which allow the thermogravimeter TGA-HP50. This apparatus employs a high-sensitivity balance with 5 g capacity and enables the measurement of a variety of gas compositions at a measurement temperature up to 800 °C and low- (vacuum) and high-pressure (15 MPa) at a static or dynamic flow regime.

The chemical treatment of CBp is one of the commonly used methods to modify and improve the functionality, pore structure, and surface area of carbonaceous materials. The chemical treatment process can alter the properties of carbonaceous materials (e.g., functional group, surface area, pore volume, and elemental composition) and increase its performance [13]. One common and inexpensive chemical used for the chemical treatment of carbonaceous materials is KOH, which was studied in many articles [14,15,16]. In all cases of carbon black activation by KOH, textural properties and adsorption capacity were improved [17,18,19].

This paper is a follow-up to an earlier article [20], which focused on ST and pyrolytic CBp characterization by means of thermogravimetry and spectroscopy, and on estimating optimal pyrolysis parameters. This paper, in turn, deals with:

- The activation of CBp prepared under the above-mentioned conditions (pyrolysis at 600 °C, heating rate of 10 °C·min^−1^), in order to achieve improvement in textural properties. Due to its great ability to improve porosity, KOH was selected as an activating agent.
- Evaluation of sorption capacity by means of gravimetric method of gas sorption for two gases, i.e., N_2_ and CO_2_, at a high relative pressure, and sorption capacity for pure cyclohexane vapor at a low relative pressure were both investigated. N_2_ was chosen as a reference gas. CO_2_ is a representative greenhouse gaseous pollutant in the air, contributing to global warming [21]. Cyclohexane is a representative waste non-polar VOC solvent from the chemical industry [22].
- Determination of specific surface area of CBp and CBa by adsorption of N_2_, CO_2_, and cyclohexane at specific experimental conditions using the sorption gravimetric method at the constant pressure. Primary data were treated by the model of Langmuir isotherms and evaluated parameters were used for specific surface area calculation.

The results of our investigation could be a useful resource for academia and the industry to deal with sorbents prepared from waste polymers for gaseous air pollutants applications.

## 2. Results and Discussion

### 2.1. Material Characterization

STs are composed of moisture (0.7 wt%), volatiles (62 wt%), fixed carbon (33 wt%), and ash (4 wt%) and the STs gross calorific have a value of 38,034 J·g^−1^ [20]. By elemental analysis, it was determined that carbon black contains 85.3% C, 0.3% H_2_, 0.3% N_2_, 2.3% S, and 6.8% O_2_. The most common types of rubber used in scrap tyres are natural, butadiene, and styrene-butadiene rubber. STs also contain a relatively small amount of oil, plasticiser (contains silicon), and metals, for example zinc [23]. Compared to other activated carbon precursors (e.g., coal, wood), STs have a lower ash and fixed carbon content [24].

**Figure 1:**
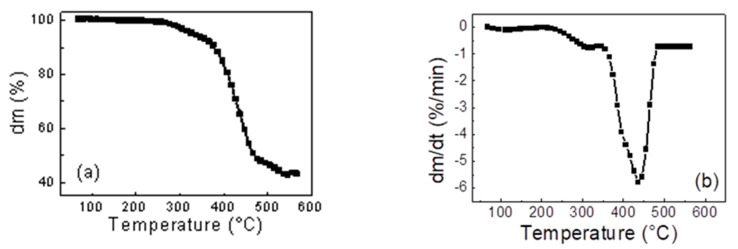
(**a**) TG curve and (**b**) DTG curve of scrap tyre (ST) pyrolyzed at 600 °C and heating rate of 10 °C·min^−1^.

The course of ST pyrolysis is documented in TG and DTG curves in Figure 1. The TG curve with a typical S-curve is shown in Figure 1a. The TG curve did not reach a zero value but only ~40 wt%. Approximately 60 wt% of volatiles are released from the sample of ST that corresponds to ~62 wt% of volatiles measured by proximate analysis and data from literature sources [24]. First, derivation of the TG curve (DTG curves) is shown in Figure 1b. From Figure 1b, it is obvious that the pyrolysis of ST starts at ~220 °C and the whole process is finished at ~500 °C. The total weight loss was again ~60 wt%. The pyrolysis of ST proceeded in two stages (Figure 1b). First, the peak was reached at ~300 °C and corresponds to the vaporization of oils, plasticizers, and additives. Second, the peak was reached at 440 °C and it refers to the rubber decomposition (natural, butadiene, and styrene-butadiene rubber). The particle, after being totally pyrolyzed, contained only fixed carbon black and inorganic matter.

**Figure 2:**
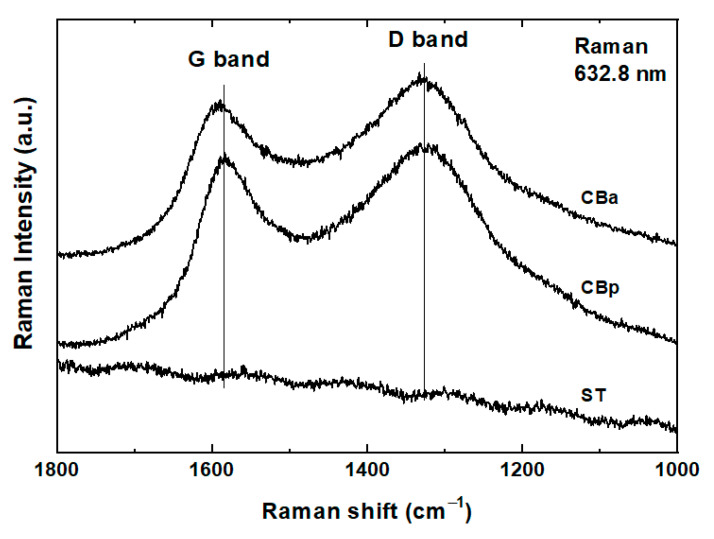
Raman spectra of scrap tyre (ST), pyrolytic carbon black (CBp), and activated carbon black (Cba).

Raman spectra of the CBp, CBa, and original ST reflect their structural order (Figure 2). As the Raman spectroscopy is a surface sensitive method, the Raman spectra of the ST sample, measured repeatedly on separated spots, do not reflect the presence of carbon black due to the not sufficiently high concentration of the carbon particles in the surface layer. The particles of the carbon black in the original ST material are evenly distributed and embedded in the rubber matrix.

The main Raman bands revealed in the spectra of both CBp and CBa are G-band at 1590 cm^−1^, “graphitic”, which is connected with the C=C stretching vibrations of any pair of sp2 sites, and D-band at 1355 cm^−1^, “disorder” band assigned to the breathing vibration of the aromatic rings, whose symmetry is broken due to their proximity to the edge of a graphite sheet, or presence of a heteroatom [25]. The Raman spectra of the CBa and CBp are virtually unchanged. The calculated ratio of areas I of the G and D bands is comparable for both carbon blacks (value I_D_/I_G_ for CBp is 1.88 and for CBa is 1.81). Thus, the activation process does not influence the carbonaceous structure of the carbon black itself. Detailed parameters of Raman peaks were published earlier in [20].

**Figure 3:**
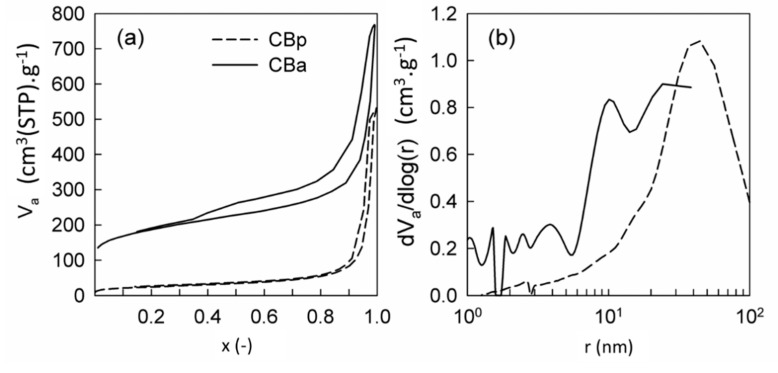
(**a**) Measured nitrogen adsorption-desorption isotherms at 77 K and (**b**) evaluated pore-size distributions of prepared carbon blacks.

Textural properties of original ST, CBp, and CBa defined from the nitrogen and krypton physisorption measurements are documented in Table 1. The progress of N_2_ adsorption-desorption isotherms and evaluated pore-size distributions of studied CBp and CBa are illustrated in Figure 3 and Figure 3b, respectively.

From Table 1, it is obvious that the original ST is nonporous and possesses a very low S_BET_ (~0.13 m^2^.g^−1^). Contrary to that, the shapes of N_2_ adsorption-desorption isotherms with hysteresis loops of produced CBp and CBa basically correspond to types II and IV+I, respectively, according to the IUPAC classification [26]. This suggests porous properties of both produced samples. CBp shows the developed predominantly macroporous structure (Figure 3b), having the S_BET_ of 88 m^2^·g^−1^. Contrary to CBp, the CBa shows, besides the irregular mesoporous-macroporous structure, also a significant amount of micropores. This feature of CBa matches its substantially improved textural properties, compared to CBp (Table 1). While CBp has the value of S_BET_ at only 88 m^2^·g^−1^, CBa possesses S_BET_ of 644 m^2^·g^−1^. V_micro_ in CBa makes up approx. 18% from the total pore volume, which is also nicely visible in Figure 3a. It can be summarized that the KOH activation of the pyrolytic carbon black led to further improvement of carbon black textural properties, resulting in an irregular mesoporous-macroporous structure including a larger amount of micropores.

Compared to the commercial carbon black, denoted N220 (114–124 m^2^·g^−1^), N330 (78–88 m^2^·g^−1^), N550 (38–46 m^2^·g^−1^), and N660 (30–40 m^2^·g^−1^) [27], the specific surface area of the produced CBp is similar to N330 and higher than N550 and N660. On the other hand, the specific surface of CBa is six times higher than all the mentioned types of commercial carbon black.

### 2.2. Sorption Experiments on Gases and Vapor

**Figure 4:**
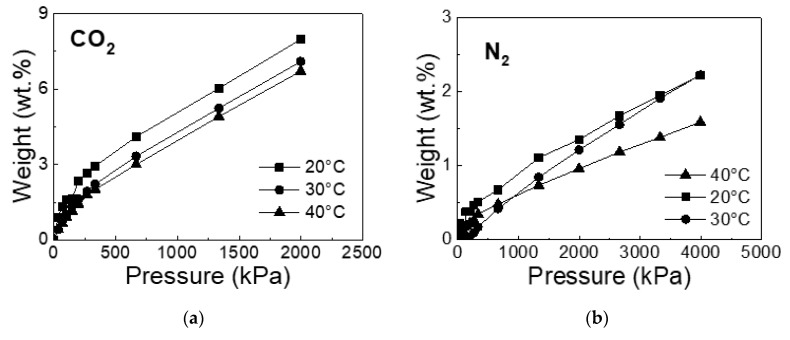
Adsorption isotherms of CO_2_ (**a**) and N_2_ (**b**) for CBp at 20, 30, and 40 °C.

The results of sorption capacity reflect the dependence of the test material on temperature and pressure. The determination of adsorption isotherms for CBp and CBa was carried out at 20, 30, and 40 °C for pure gases (N_2_ and CO_2_) and pressure range 30–4000 kPa for N_2_ and 30–2000 kPa for CO_2_. For cyclohexane vapors, the adsorption isotherms were measured at 30 °C when the low pressures of saturated vapor were applied (10.5 kPa). The maximum gas uptake of CO_2_ was registered at the lowest temperature and the highest pressure, which is in good agreement with general assumptions about adsorption processes onto solid surfaces [28], which is documented in Figure 4.

The amount of adsorbed gas at a maximum pressure of 2000 kPa (for CO_2_) and 4000 kPa (for N_2_) is documented in Table 2 and Table 3. It can be seen that the adsorbed amount of CO_2_ is five times higher than the adsorbed amount of N_2_ for non-activated CBp. Comparing activated and non-activated carbon blacks, the sorption capacity of CBa is two times higher than that of CBp for CO_2_ at 20 °C. It can be concluded that the sorption capacity of carbon black depends on both the surface character and the porous structure of adsorbents. A generally better sorption capacity of CBp for CO_2_ than for N_2_ may be explained by the acid character of CO_2_ and carbon black surface. The significantly improved sorption capacity of CBa for CO_2_ than of CBp may be attributed to the increased surface basicity of CBa caused by the KOH activation [29]. The activation of CBp also significantly improved its porous structure, developing the microporosity as well as the meso-macroporosiy, thus enlarging the carbon black surface area which is enforced in sorption.

Table 2 and Table 3 also compare experimental and literature data. According to [16], carbon black was prepared by pyrolysis at 550 °C from beech and a mixture of beech and oak (denoted Beech NA and Beech/Oak NA). In the same temperature regime, original biomass samples were also activated with K_2_CO_3_ (ratio 3:1—K_2_CO_3_:sample) and then pyrolyzed (denoted as Beech A and Beech/Oak A).

Based on sorption experimental results, it is certain that the sorption capacity is dependent on activation and precursors of the sample. It can be seen that better sorption capacities (for CO_2_ and N_2_) were obtained on carbon black prepared from biomass precursors.

The sorption capacity of CBa for CO_2_ is comparable to non-activated Beech NA. The best results were obtained for beech activated (Beech A), where approx. 7% of N_2_ and approx. 25% of CO_2_ were adsorbed. Compared to the literature [16], the sorption capacity:

- of CBp is up to three times lower than for Beech NA and Beech/Oak NA for N_2_ adsorption,
- of CBp is up to two times lower than for Beech NA for CO_2_ adsorption,
- of CBa is slightly higher than Beech NA for CO_2_ adsorption,
- of CBa is up to two times lower than for Beech A for CO_2_ adsorption.

The Langmuir isotherm [30] was used for evaluation of sorption uptake of CO_2_ and cyclohexane at different temperatures on activated CBa and non-activated CBp. The linearized model of Langmuir isotherm is expressed in Equation (1), where p is vapor pressure, p_0_ is saturated vapor pressure, n_A_ is the adsorbated amount of vapor, n_m_ is the monolayer capacity of the adsorbent, and K is the constant related to the energy of adsorption.

(1) $$\frac{p/p_{0}}{n_{A}}=\frac{1}{n_{m}}\frac{p}{p_{0}}+\frac{1}{{\mathrm{Kn}}_{m}p_{0}}$$

**Figure 5:**
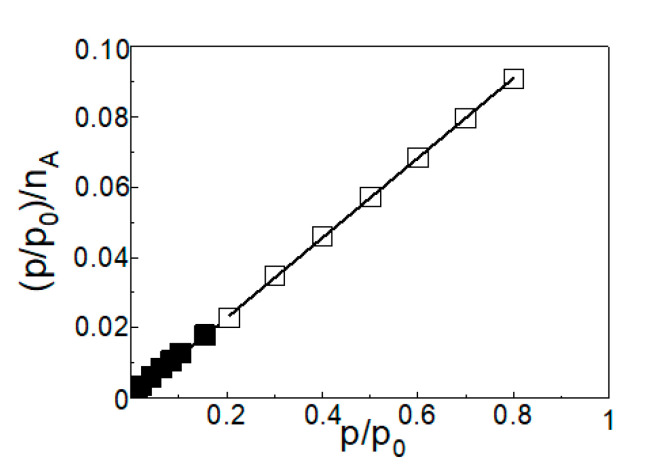
Linearized Langmuir adsorption isotherm of cyclohexane on CBa at 30 °C. Solid points are up to the relative pressure p/p_0_ of 0.2, open points are over the relative pressure p/p_0_ of 0.2.

The linearized Langmuir isotherm of cyclohexane on activated CBa at a temperature of 30 °C is shown in Figure 5.

The monolayer capacity of the adsorbent (n_m_), the constant related to the energy of adsorption (K), and the coefficient of determination (R^2^) were calculated and can be seen in Table 4.

Linearized data up to the relative pressure value p/p_0_ of 0.2 are plotted as a solid point, data above the relative pressure value p/p_0_ of 0.2 are plotted as an open point.

From the shape of Langmuir isotherms of CO_2_ and cyclohexane, an increase in CO_2_ gas uptake on activated CBa is visible up to p/p_0_ = 0.08. On the other hand, gas uptake in cyclohexane on activated carbon CBa is most evident up to p/p_0_ = 0.15. Further increasing the relative pressure leads to an increase in gas uptake of CO_2_ by ~0.04 g·g^−1^ and of cyclohexane by ~0.03 g·g^−1^.

The monolayer capacity n_m_ of the adsorbent decrease in the order: CBa for cyclohexane ˃ CBa for CO_2_ ˃ CBp for cyclohexane ˃ CBp for CO_2_ ˃ CBp for N_2_, is evident from Table 4. The adsorption of N_2_ is almost negligible compared with values of CO_2_ and cyclohexane. As we expected, the sorption capacity is greater for activated CBa than for non-activated carbon CBp for both gases/vapors. The coefficient of determination shows that the Langmuir isotherm model fits the data very well.

The evaluated monolayer capacity n_m_ of CBp and CBa was used to calculate the apparent specific surface areas of adsorbents. The cross-sectional areas of:

- N_2_ was taken as 0.162 nm^2^ [31].
- CO_2_ was taken as 0.187 nm^2^ [31].
- Cyclohexane was taken as 0.431 nm^2^ [30].

For the adsorption of CO_2_, N_2_, and cyclohexane, the surface area for both CBp and CBa was calculated based on the parameters of the Langmuir isotherm. The assumption of no presence of capillary condensation was applied and determined values of surface areas were compared with those of standard nitrogen physisorption measurements, using the BET method ( Table 1 and Table 4).

Physical properties and three-dimensional spacing of adsorbate molecules are crucial parameters affecting sorption. Calculated specific areas of samples decreased in this order: cyclohexane > CO_2_ > N_2_. The specific surface area determined by the nitrogen S-BET method is closest to the specific surface areas obtained by CO_2_ for CBp and by cyclohexane for CBa. Variations between S-BET and the specific surface from Langmuir isotherm can be explained by the different methodologies/theoretical assumptions of the applied models used for evaluation as well as by the different nature of the used adsorptive.

**Figure 6:**
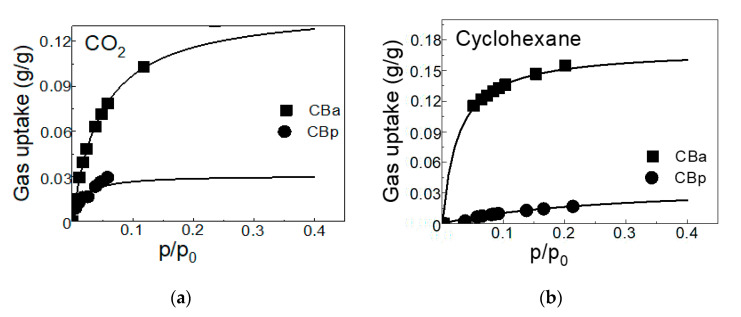
Adsorption and Langmuir isotherms of CO_2_ (**a**) for CB_p_ and CBa at 20 °C and cyclohexane (**b**) vapor for CBp and for CBa at 30 °C.

It is obvious from Table 1 that the activation of CBp essentially improves the textural properties (i.e., mesopore surface area, micropore volume, and total pore volume) of the final carbonaceous product (CBa), which positively affects the adsorption of gases. Activation with KOH at 800 °C leads to the active carbon samples of a well-developed porous structure with predominant micropores [1]. This feature is in agreement with the literature [32,33]. The activation of carbon black prepared by pyrolysis of waste ST with alkali, increased the sorption capacity, with a decrease in ash content. For CBa, the uptake of CO_2_ is approximately three times higher and the uptake of cyclohexane is ~10 times higher than for CBp (Figure 6). The affinity of CBa to cyclohexane vapors is higher by 3% than to CO_2_.

**Figure 7:**
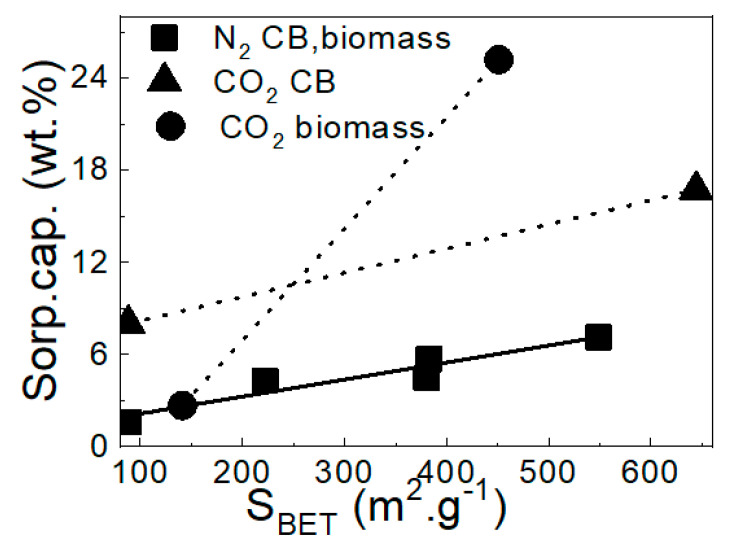
Linear relationship between the specific surface area (S_BET_) and sorption capacity of N_2_ at the pressure 4000 kPa and of CO_2_ at the pressure 2000 kPa for CBa and biomass [16].

Many studies have revealed that the specific surface area of carbonaceous adsorbents is primarily responsible for physical adsorption of organic compounds. Therefore, the higher surface area of activated carbon had larger adsorption rate constants implying that physical adsorption may be the dominating mechanism [34]. The positive relationship between surface area and adsorption capacity of N_2_ was investigated, which is illustrated in Figure 7. According to the literature [29,35] a larger total pore volume provides more active sites for interaction between CO_2_ and the carbon black. In this article, the CO_2_ adsorption capacity has a linear correlation with the micropore surface area and also BET surface area. In our case, the sorption capacity of N_2_ and carbon black also shows a linear relationship. For these reasons, we could also assume a positive relationship between the sorption capacity of CO_2_ and carbon black. Therefore, we connect points for sorption capacities for CO_2_ with a dotted line in Figure 7. It can be concluded that the adsorption of N_2_ depends only on the size of the specific surface area. Adsorption is not affected by the nature (ST or biomass) of the carbon. On the other hand, adsorption of CO_2_ depends on the value of the specific surface area and also on the nature of the carbon. For lower S_BET_ values (<240 m^2^/g), the sorption capacity is higher for CBa derived from ST, conversely for higher S_BET_ values (>240 m^2^/g), the sorption capacity is higher for the activated carbon prepared from biomass [16].

## 3. Experimental Part

### 3.1. Material Preparation

Waste scrap tyres (denoted as STs) (undefined mixture, Moravia-Silesian region, Czech Republic) were collected from passenger cars from car wrecker. STs were cut into smaller pieces and steel wires were removed by hand. After that, STs were crushed with a Testchem LMN-100 mill into a smaller part and then sampled through assay sieves under 1 mm. The sieves are made by Preciselekt and comply with the ISO 3310 standard.

Pyrolytic carbon black (denoted as CBp) was prepared using thermogravimeter TG-DTA NETZSCH STA 409 EP (NETZSCH, Selb, Germany). Experiments were conducted in big 5 mL crucibles from aluminum oxide in a dynamic inert argon atmosphere (flow rate of 100 cm^3^·min^−1^) at 600 °C for 3 min with a heating rate of 10 °C min^−1^.

Ten grams of prepared CBp were mixed in the polyethylene bottle with a KOH (Merck, Kenilworth, NJ, USA) solution (121 g/100 mL) at a 1:3 mass ratio (sample:KOH). This mixture, with a small amount of tenside (commercial dish detergent, added in order to increase the wettability of CBp by KOH) was left at 25 °C for 1 day. The sample impregnated with KOH was loaded to a porcelain combustion boat. Activation took place in a quartz tube with a 25 mm inner diameter in a dynamic inert N_2_ atmosphere (flow rate 200 mL min^−1^). Activation was performed at 800 °C for 30 min in a tube furnace LT 50/300/13 (LAC, Židlochovice, Czech Republic). After activation and cooling, the sample was washed on a glass fiber filter with distilled water to achieve a neutral pH. The washed activated carbon was dried at 105 °C over night. The activated sample was denoted as CBa.

### 3.2. Material Characterization

Raman spectra excited in the visible range with an HeNe 633 nm laser were measured on powder samples. A microscope with an objective 50× magnification was used to focus the laser beam on the sample. The scattered light was analyzed by a spectrograph with a holographic grating of 1800 lines mm^–1^. A Peltier cooled CCD detector (576 × 384 pixels) registered the dispersed light.

Nitrogen and krypton physisorption measurements at 77 K were performed by using the ASAP2020 physisorption instrument (Micromeritics, Nocross, GA, USA) and NOVA2000e (Quanta chrome Instruments, Boynton Beach, FL, USA). Prior to the physisorption measurements, the materials were degassed at 105 °C under a vacuum less than 1 Torr (133 Pa) for 14 h. The following textural properties were evaluated: Specific surface area, S_BET_, from the adsorption isotherm of nitrogen or krypton for the p/p_0_ = 0.05–0.25 range using the standard Brunauer-Emmett-Teller (BET) procedure [36], and mesopore surface area, S_meso_, and micropore volume, V_micro_, evaluated by the t-plot method [37]. Pore-size distribution (pore radius 10^0^–10^2^ nm) was determined from the adsorption branch of nitrogen adsorption-desorption isotherm by the advanced Barrett-Joyner-Halenda (BJH) method [38,39]. The Lecloux-Pirard standard isotherm [40,41] was used for the t-plot, as well as for the pore-size distribution evaluations. The total pore volume, V_total_, was determined as the adsorbed volume of nitrogen at relative pressure p/p_0_ = 0.990.

The proximate analysis according to the standard ASTM D7582 (LECO, TGA 701) was done for raw scrap tyres.

**Figure 8:**
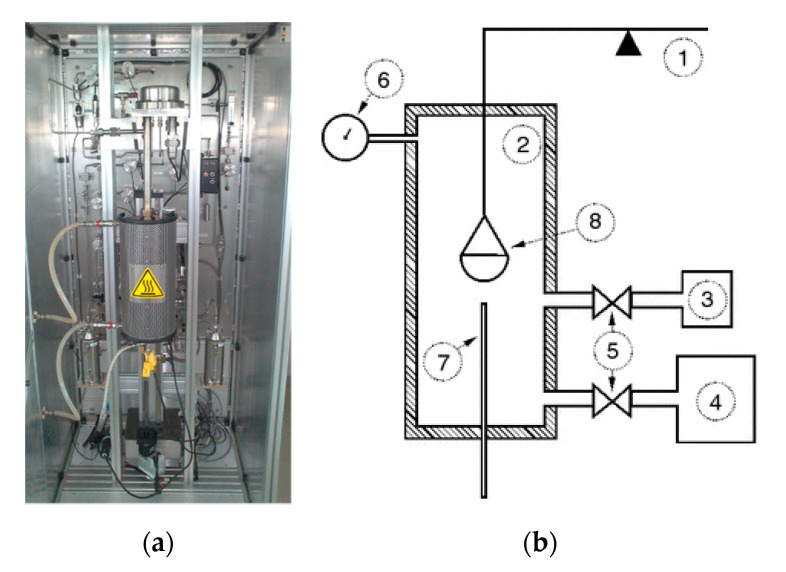
Photo (**a**) and scheme (**b**) of essential parts of operating unit of the thermogravimeter TGA-HP50 for sorption experiments: 1: High-sensitivity balance, 2: Tempered measuring cell, 3: VOC container, 4: Vacuum accessories, 5: Automatic valves, 6: Pressure transducer, 7: Thermocouple, 8: Measuring can with the tested sorbent.

The tempered experimental cell where the solid sorbent takes place, high-sensitivity balance, pressure transducer, special container for VOC in a liquid state, vacuum accessories, and a set of automatic valves are the essential parts of the thermogravimeter TGA-HP50 was employed at the sorption experiments. The operating principle and connections of the parts are illustrated in Figure 8. Inside the tempered cell there is a solid sample placed into the glass pan, which is connected to the high-sensitivity of 0.5 μg balance by a thin fiber. The sample quantity was about 35 mg. Firstly, the cell is evacuated down to the pressure below 0.1 torr. After evacuation, the drying period of the experiment starts. The sample is heated up to 120 °C and kept at such temperature for a certain time. Then, the sample is cooled down to the experimental temperature (20, 30, or 40 °C) and the low pressure (below 0.1 torr).

The adsorption part of the experiments starts when the automatic valve separating the measuring cell and valve for the gas (N_2_ or CO_2_) or container with a liquid VOC (cyclohexane) becomes open. The adsorption branch of the experiment consists of several constant pressure steps, which are organized with upward trends to the maximum pressure (0.03–2 MPa for CO_2_; 0.03–4.5 MPa for N_2_, and to the saturated pressure of cyclohexane). The mass of the adsorbent sample is measured permanently during all experimental periods. The uncertainty of measurements is when the mass of the sample is stable for 5 min ± 0.5 mass%. When the last specified pressure step at a certain temperature is complied, the experiment is finished and the pressure inside the measuring cell is gently increased to the ambient pressure.

The measured quantities were change of mass of the solid sample (g), time (min), temperature (°C), and pressure (kPa) inside the measuring cell. The model of Langmuir isotherm was used for the determination of adsorption curves of CO_2_ and cyclohexane vapor.

## 4. Conclusions

This paper deals with the preparation and chemical activation of carbon black prepared by pyrolysis of waste scrap tyres. Non-activated pyrolytic carbon black, CBp, was prepared by pyrolysis of waste scrap tyres at the temperature of 600 °C and heating rate of 10 °C·min^−1^ for 3 min in an inert argon. In addition to that, it was activated by a KOH solution at the temperature of 800 °C to reach the activated form of carbon black, CBa. The activation of pyrolytic carbon black, CBp, did not affect its rate of graphitization, but it significantly affected the porous structure of activated CBa. The specific surface area increased from 88 m^2^·g^−1^ for non-activated CBp to 644 m^2^·g^−1^ for activated CBa, in the activated CBa the irregular mesoporous-macroporous structure with a significant amount of micropores was formed due to activation.

The sorption capacity of non-activated CBp and activated CBa was investigated by inert gas—N_2_, greenhouse gas—CO_2_, and non-polar vapor-cyclohexane at different temperatures and pressures. The sorption capacity depends on the type of gas/vapor adsorbed and also on the character of the adsorbents. The adsorbed amount of CO_2_ at our chosen experimental conditions was five times higher than the adsorbed amount of N_2_ for CBp. The uptake of CO_2_ was three times higher and for cyclohexane ten times higher for activated CBa than for non-activated CBp at 20 °C for CO_2_ and at 30 °C for cyclohexane.

Experimental data were also treated by the linearized model of Langmuir isotherm, surface areas were calculated and compared with values determined from the linearized form of BET isotherm. In terms of closeness with the standard BET method, CO_2_ appears to be the most suitable adsorbent for non-activated CBp (78 m^2^·g^−1^) and cyclohexane for activated CBa (526 m^2^·g^−1^).

From the linear relationship between the surface area and the sorption capacity it can be summarized that the N_2_ adsorption depends only on the specific surface area and the nature of the carbon does not affect it.

It can be concluded that the production of activated carbon black from waste scrap tyres by pyrolysis shows two positive aspects from the view point of environmental protection. Firstly, the amount of waste landfilled ST, which represents a long-term ecological burden for the environment may be reduced, and secondly, a valuable solid product—activated carbon black with good sorption ability—is produced. In spite of the fact that non-activated CBp and activated CBa produced from ST do not surpass the carbon produced from biomass, the chemical activation of non-activated CBp from ST resulted in an improvement of the adsorption capacity of activated CBa.

## Figures and Tables

**Table 1:** Textural properties of original scrap tyre and produced carbon blacks.

	S_BET_	S_meso_	V_micro_	V_total_	V_micro_	V_total_
	/m^2^·g^−1^		/mm^3^_lig_·g^−1^		/cm^3^(STP)·g^−1^	
ST *	0.13	0	0	0	0	0
CBp	88	56	17	691	11	447
CBa	644	265	211	1186	136	767

* The specific surface area of the material was determined by Kr physisorption at 77 K. S_meso_: Mesopore surface area; V_micro_: Micropore volume; V_total_: Total pore volume.

**Table 2:** Results of sorption capacity in the environment of CO_2_ at a pressure of 2000 kPa, comparison of experimental and literature data [16].

Adsorbed Amount of CO_2_	CBp	CBa	Beech NA *	Beech A *	Beech/Oak NA *	Beech/Oak A *
	/wt%					
t = 20 °C	8.00	16.72	14.33	25.59	not meas.	not meas.
t = 30 °C	7.01	not meas.	13.68	24.55	not meas.	not meas.
t = 40 °C	6.71	not meas.	13.52	24.59	not meas.	not meas.

* Ref. [16], not meas.: Not measured.

**Table 3:** Results of sorption capacity in the environment of N_2_ at a pressure of 4000 kPa, comparison of experimental and literature data [16].

Adsorbed Amount of N_2_	CBp	CBa	Beech NA *	Beech A *	Beech/Oak NA *	Beech/Oak A *
	/wt%					
t = 20 °C	2.23	not meas.	5.69	7.13	4.29	4.46
t = 30 °C	1.84	not meas.	5.59	6.78	4.36	6.32
t = 40 °C	1.59	not meas.	5.67	7.37	4.46	6.62

* Ref. [16], not meas.: Not measured.

**Table 4:** Parameters of the Langmuir isotherm with the coefficient of determination at 20 °C for N_2_ and CO_2_ and at 30 °C for cyclohexane.

	CBp				CBa			
	n_m_	K	R^2^	S	n_m_	K	R^2^	S
	/g·g^−1^	/Pa^−1^		/m^2^·g^−1^	/g·g^−1^	/Pa^−1^		/m^2^·g^−1^
N_2_	0.0027	392.625	0.9466	9.3	not meas.	not meas.	not meas.	not meas.
CO_2_	0.0307	8.366	0.9150	79	0.1432	2.824	0.9977	367
Cyclohexane	0.0383	493.000	0.9774	118	0.1706	5.169	0.9950	526

Not meas.: Not measured.

## References

[1] Hofman M., Pietrzak R. (2011). Adsorbents obtained from waste tires for NO_2_ removal under dry conditions at room temperature. Chem. Eng. J..

[2] Pantea D., Darmstadt H., Kaliaguine S., Roy C. (2003). Heat-treatment of carbon blacks obtained by pyrolysis of used tires. Effect on the surface chemistry, porosity and electrical conductivity. J. Anal. Appl. Pyrolysis.

[3] Teng H.S., Serio M.A., Wojtowicz M.A., Bassilakis R., Solomon P.R. (1995). Reprocessing of used tires into activated carbon and other products. Ind. Eng. Chem. Res..

[4] Leung D.Y.C., Wang C.L. (1998). Kinetic study of scrap tyre pyrolysis and combustion. J. Anal. Appl. Pyrolysis.

[5] McCabe W.L. (2005). Unit Operations of Chemical Engineering.

[6] Rouquerol J. (1998). Adsorption by powders and porous solids. Principles, Methodology and Applications.

[7] Wankat P.C. (2011). Separation Process Engineering: Includes Mass Transfer Analysis.

[8] Seader J.D. (2011). Separation Processprinciples Chemical and BiochemicalOperations.

[9] Xi K., Chen B.A., Li H.L., Xie R.S., Gao C.L., Zhang C., Kumar R.V., Robertson J. (2015). Soluble polysulphide sorption using carbon nanotube forest for enhancing cycle performance in a lithium-sulphur battery. Nano Energy.

[10] Liu S.Y., Mei L.F., Liang X.L., Liao L.B., Lv G.C., Ma S.F., Lu S.Y., Abdelkader A., Xi K. (2015). Anchoring Fe3O4 nanoparticles on carbon nanotubes for microwave-induced catalytic degradation of antibiotics. ACS Appl. Mater. Interfaces.

[11] Pal A., Uddin K., Saha B.B., Thu K., Kil H.S., Yoon S.H., Miyawaki J. (2020). A benchmark for CO_2_ uptake onto newly synthesized biomass-derived activated carbons. Appl. Energy.

[12] Gensterblum Y., van Hemert P., Billemont P., Busch A., Charriere D., Li D., Krooss B.M., de Weireld G., Prinz D., Wolf K. (2009). European inter-laboratory comparison of high pressure CO_2_ sorption isotherms. I: Activated carbon. Carbon.

[13] Wahi R., Zuhaidi N.F.Q., Yusof Y., Jamel J., Kanakaraju D., Ngaini Z. (2017). Chemically treated microwave-derived biochar: An overview. Biomass Bioenergy.

[14] Pal A., Thu K., Mitra S., El-Sharkawy I.I., Saha B.B., Kil H.S., Yoon S.H., Miyawaki J. (2017). Study on biomass derived activated carbons for adsorptive heat pump application. Int. J. Heat Mass Transf..

[15] Hofman-Bieniek M., Pietrzak R. (2017). Thermal study of adsorbents prepared from waste tyres. Environ. Eng. Manag. J..

[16] Smatanová N., Koutník I., Večeř M. (2013). Effect of chemical activation on sorption characteristics of selected wood samples. Geosci. Eng..

[17] Li D.W., Ma T.F., Zhang R.L., Tian Y.Y., Qiao Y.Y. (2015). Preparation of porous carbons with high low-pressure CO2 uptake by KOH activation of rice husk char. Fuel.

[18] Martinez-Escandell M., de Castro M.M., Molina-Sabio M., Rodriguez-Reinoso F. (2013). KOH activation of carbon materials obtained from the pyrolysis of ethylene tar at different temperatures. Fuel Process. Technol..

[19] Shen Z.M., Xue R.S. (2003). Preparation of activated mesocarbon microbeads with high mesopore content. Fuel Process. Technol..

[20] Mikulova Z., Sedenkova I., Matejova L., Vecer M., Dombek V. (2013). Study of carbon black obtained by pyrolysis of waste scrap tyres. J. Therm. Anal. Calorim..

[21] (2020). Data and Statistics.

[22] Ojala S., Pitkaaho S., Laitinen T., Koivikko N.N., Brahmi R., Gaalova J., Matejova L., Kucherov A., Paivarinta S., Hirschmann C. (2011). Catalysis in VOC abatement. Top. Catal..

[23] Januszewicz K., Klein M., Klugmann-Radziemska E., Kardas D. (2016). Thermogravimetric analysis/pyrolysis of used tyres and waste rubber. Physicochem. Probl. Miner. Process.

[24] Bansal R.C., Goyal M. (2005). Activated Carbon Adsorption.

[25] Darmstadt H., Pantea D., Sümmchen L., Roland U., Kaliaguine S., Roy C. (2000). Surface and bulk chemistry of charcoal obtained by vacuum pyrolysis of bark: Influence of feedstock moisture content. J. Anal. Appl. Pyrolysis.

[26] Sing K.S.W., Everett D.H., Haul R.A.W., Moscou L., Pierotti R.A., Rouquerol J., Siemieniewska T. (1985). Reporting physisorption data for gas solid systems with special reference to the determination of surface-area and porosity (recommendations 1984). Pure Appl. Chem..

[27] Korenova Z., Haydary J., Annus J., Markos J., Jelemensky L. (2008). Pore structure of pyrolyzed scrap tires. Chem. Pap..

[28] Smutná J., Ciahotný K., Ubrá O., Vrbová V., Machač P., Pilař L., Vitvarová M. (2016). Adsorpce CO_2_ ze spalin elektráren na pevných sorbentech. Paliva.

[29] Creamer A.E., Gao B., Wang S.S. (2016). Carbon dioxide capture using various metal oxyhydroxide-biochar composites. Chem. Eng. J..

[30] Vecer M., Spitova B., Koutnik I. (2015). Determination of specific surface of activated mesocarbons by sorption of organic vapors. J. Therm. Anal. Calorim..

[31] Morenocastilla C., Carrascomarin F., Lopezramon M.V. (1995). Micropore structure of activated carbons prepared from a spanish subbituminous coal studied by CO_2_, benzene, and cyclohexane adsorption. Langmuir.

[32] Barnakov C.N., Khokhlova G.P., Vershinin S.N., Samarov A.V. (2015). Carbon sorbents produced from truck tires. Coke Chem..

[33] Rambau K.M., Musyoka N.M., Manyala N., Ren J., Langmi H.W. (2018). Mechanochemical approach in the synthesis of activated carbons from waste tyres and its hydrogen storage applications. Mater. Today Proc..

[34] Zhang X.Y., Gao B., Fang J.N., Zou W.X., Dong L., Cao C.C., Zhang J., Li Y.C., Wang H.L. (2019). Chemically activated hydrochar as an effective adsorbent for volatile organic compounds (VOCs). Chemosphere.

[35] Dissanayake P.D., You S.M., Igalavithana A.D., Xia Y.F., Bhatnagar A., Gupta S., Kua H.W., Kim S., Kwon J.H., Tsang D.C.W. (2020). Biochar-based adsorbents for carbon dioxide capture: A critical review. Renew. Sustain. Energy Rev..

[36] Brunauer S., Emmett P.H., Teller E. (1938). Adsorption of Gases in Multimolecular Layers. J. Am. Chem. Soc..

[37] de Boer J.H., Lippens B.C., Linsen B.G., Broekhoff J.C.P., van den Heuvel A., Osinga T.J. (1966). Thet-curve of multimolecular N2-adsorption. J. Colloid Interface Sci..

[38] Barrett E.P., Joyner L.G., Halenda P.P. (1951). The determination of pore volume and area distributions in porous substances. I. computations from nitrogen isotherms. J. Am. Chem. Soc..

[39] Roberts B.F. (1967). A procedure for estimating pore volume and area distributions from sorption isotherms. J. Colloid Interface Sci..

[40] Lecroux A. (1979). The importance of standard isotherms in the analysis of adsorption isotherms for determining the porous texture of solids. J. Colloid Interface Sci..

[41] Schneider P. (1995). Adsorption isotherms of microporous-mesoporous solids revisited. Appl. Catal. A Gen..

